# Gene Updater: a web tool that autocorrects and updates for Excel misidentified gene names

**DOI:** 10.1038/s41598-022-17104-3

**Published:** 2022-07-26

**Authors:** Clara W. T. Koh, Justin S. G. Ooi, Gabrielle L. C. Joly, Kuan Rong Chan

**Affiliations:** Programme in Emerging Infectious Diseases, Duke-NUS Medical School, Singapore, 169857 Singapore

**Keywords:** Software, Data publication and archiving, Microarrays, Software

## Abstract

Opening and processing gene expression data files in Excel runs into the inadvertent risk of converting gene names to dates. As pathway analysis tools rely on gene symbols to query against pathway databases, the genes that are converted to dates will not be recognized, potentially causing voids in pathway analysis. Molecular pathways related to cell division, exocytosis, cilium assembly, protein ubiquitination and nitric oxide biosynthesis were found to be most affected by Excel auto-conversion. A plausible solution is hence to update these genes and dates to the newly approved gene names as recommended by the HUGO Gene Nomenclature Committee (HGNC), which are resilient to Excel auto-conversion. Herein, we developed a web tool with Streamlit that can convert old gene names and dates back into the new gene names recommended by HGNC. The web app is named Gene Updater, which is open source and can be either hosted locally or at https://share.streamlit.io/kuanrongchan/date-to-gene-converter/main/date_gene_tool.py. Additionally, as Mar-01 and Mar-02 can each be potentially mapped to 2 different gene names, users can assign the date terms to the appropriate gene names within the Gene Updater web tool. This user-friendly web tool ensures that the accuracy and integrity of gene expression data is preserved by minimizing errors in labelling gene names due to Excel auto-conversions.

## Introduction

When gene expression datasets are opened with Excel under default settings (Microsoft Corp., Redmond, WA), a recurring problem where gene names are converted to dates occurs. Similarly, if gene names are copied from another application (e.g. text processors) and pasted into an Excel spreadsheet without specifying cell formatting, conversion of gene names to dates can occur^1^. While Excel is popular and widely used in data analysis, these auto-conversions can affect pathway enrichment analysis, as many of the pathway enrichment tools such as Enrichr^2^, Gene set enrichment analysis (GSEA)^3,4^ and Ingenuity Pathway Analysis^5^ rely on gene symbols to query against pathway databases such as Gene Ontology^6,7^ and Reactome^8^. As dates are not recognized by these pathway databases, this can result in voids in pathway enrichment analysis. For instance, septins (e.g. SEPT1), which are involved in cell division, are internally converted to SEP-01 in Excel, which cannot be recognized by other databases. This problem has become so rampant that approximately one-fifth of the published papers with supplementary Excel gene lists contain erroneous gene name conversions^9,10^. As many of these datasets are frequently accessed by other data scientists, such errors may be carried over to other scientific publications, resulting in further distortion of downstream data analysis.

To tackle this issue, the HUGO Gene Nomenclature Committee (HGNC) announced in 2017 to update the gene names that may be unintentionally converted to dates in Excel files^11^. This movement was well-received by researchers and data scientists, as changing to the updated gene names would allow sharing of gene expression data without worrying about the automatic conversion of gene symbols to dates in Excel. However, at present, most of the published gene expression data are not updated to the newly approved gene names, especially in the microarray datasets. We thus developed a Gene Updater web tool that allows researchers to convert the previous gene names to the newly approved gene names recommended by HGNC. Moreover, if the gene names are unintentionally converted to dates by Excel, the web tool allows researchers to rectify these terms back to the correct gene names. We believe that these efforts will facilitate gene expression data sharing between researchers who may be working on different analytics platforms.

### Codes availability and issue reporting

The Gene Updater webtool is publicly available at: https://share.streamlit.io/kuanrongchan/date-to-gene-converter/main/date_gene_tool.py. The code was written with the Python programming language (https://www.python.org/) and the web tool is made with Streamlit (https://www.streamlit.io). To run the app locally, several freely available packages are required: pandas, numpy, regex, inflect, dateparser, streamlit, streamlit-tags, openpyxl, xlrd, and XlsxWriter. The recommended versions are as follows: pandas >  = 1.2.5, numpy >  = 1.19.5, regex >  = 2021.8.3, inflect >  = 5.3.0, dateparser >  = 1.1.0, streamlit >  = 1.8.1, streamlit-tags >  = 1.2.8, openpyxl >  = 3.0.9, xlrd >  = 2.0.1, XlsxWriter >  = 3.0.2. The MIT licence version 0.1.0 is also added to the source package on GitHub as an open source licence.

The up-to-date codes and new releases will be made available on GitHub, including the step-by-step protocol information on running the app locally: https://github.com/kuanrongchan/date-to-gene-converter (Zenodo; https://doi.org/10.5281/zenodo.6845701). This page can also be used to communicate any issues, queries, or request features. Otherwise, users can contact the developers via email.

### Overview of Gene Updater

**Figure 1:**
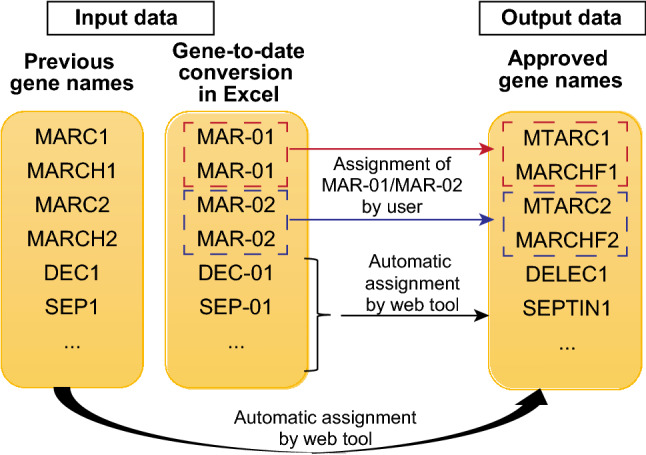
Schematic of Gene Updater. If old gene names are provided, these genes will be automatically converted to the updated approved gene names. If dates are provided, all genes, with the exception of MAR-01 and MAR-02 will be converted to the new approved gene names. For MAR-01 and MAR-02, users can assign the genes to either MTARC1, MARCHF1, MTARC2 or MARCHF2 within Gene Updater.

Users can directly upload data from Excel spreadsheets or csv files containing gene names into Gene Updater (https://share.streamlit.io/kuanrongchan/date-to-gene-converter/main/date_gene_tool.py). The old gene names will be automatically updated to the new gene names with Gene Updater. If genes were converted to dates by Excel, these dates will also be automatically converted to the updated gene names, except for Mar-01 and Mar-02 as these dates can be potentially mapped to more than one gene (Fig. 1). The conversion of Mar-01 to either MTARC1 or MARCHF1, as well as Mar-02 to either MTARC2 or MARCHF2 can be assigned by the user within the Gene Updater web tool. The output is an Excel data file containing the updated HUGO gene names which can be downloaded for further downstream analysis.

### Data input

The user interface starts with a file uploader that enables users to upload their .csv or .xlsx file(s). Multiple files can also be uploaded, as long as the first column contains the gene names. If the gene file contains Mar-01 and Mar-02, we encourage having an identifier (e.g. gene description) on the second column so that the identities of MARCH1/MARC1 and MARCH2/MARC2 can be easily resolved with the Gene Updater. Users do not have to remove the other data columns in their Excel or csv files to use the web tool. The checkbox located at the sidebar allows users to inspect that the correct data file is uploaded. To demonstrate the features of the app, a demo dataset containing Excel converted gene terms, gene descriptions and numeric values is pre-loaded if no data files are uploaded into Gene Updater.

### Identity and characteristics of genes that are changed by Gene Updater

**Figure 2:**
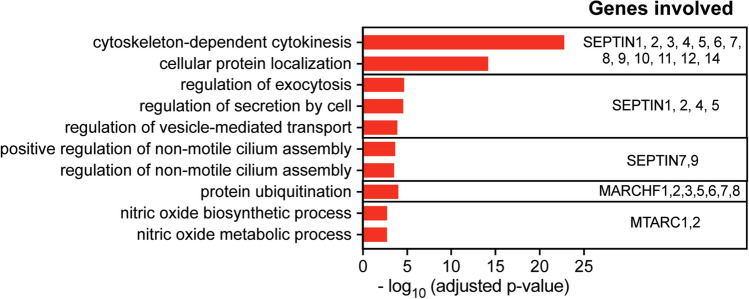
Top 10 enriched pathways based on genes that are frequently converted to dates in Excel. Genes were analysed against the GO Biological Processes database with Enrichr, and all presented pathways had adjusted *p*-values < 0.05.

The human gene names that are converted to dates in Excel, with their updated approved gene names and descriptions are detailed in Table 1. With the exception for Mar-01 and Mar-02, all other genes modified by Excel can be mapped to a unique HUGO gene (Table 1). To examine the impact of omitting these genes due to Excel conversions on pathway analysis, we examined the biological processes modulated by these genes. Pathway enrichment analysis against the Gene Ontology (GO) Biological Processes database^6,7^ highlighted that these genes play a critical role in cell division, exocytosis, cilium assembly, ubiquitination, and nitric oxide biosynthesis (Fig. 2). Specifically, SEPTIN1-14 is encoded for pathways related to cytoskeleton-dependent cytokinesis, some of which are also involved in other specialised functions such as exocytosis, secretion and cilium assembly. On the other hand, MARCH1-8 are involved in protein ubiquitination whereas MTARC1 and MTARC2 are involved in nitric oxide biosynthesis and metabolic processes (Fig. 2). Overall, our results highlight the voids in pathway enrichment analyses if these gene names are auto-converted to dates in Excel.

**Table 1:** Human gene names that are most frequently converted to dates in Excel. The respective updated gene name and gene description is also provided.

Previous gene name	Excel Date Conversion	HUGO Gene	Entrez gene description (Homo sapiens)
DEC1	Dec-01	DELEC1	Deleted in oesophageal cancer 1
MARC1	Mar-01	MTARC1	Mitochondrial amidoxime reducing component 1
MARCH1	Mar-01	MARCHF1	Membrane associated ring finger 1
MARCH2	Mar-02	MARCHF2	Membrane associated ring finger 2
MARC2	Mar-02	MTARC2	Mitochondrial amidoxime reducing component 2
MARCH3	Mar-03	MARCHF3	Membrane associated ring finger 3
MARCH4	Mar-04	MARCHF4	Membrane associated ring finger 4
MARCH5	Mar-05	MARCHF5	Membrane associated ring finger 5
MARCH6	Mar-06	MARCHF6	Membrane associated ring finger 6
MARCH7	Mar-07	MARCHF7	Membrane associated ring finger 7
MARCH8	Mar-08	MARCHF8	Membrane associated ring finger 8
MARCH9	Mar-09	MARCHF9	Membrane associated ring finger 9
MARCH10	Mar-10	MARCHF10	Membrane associated ring finger 10
MARCH11	Mar-11	MARCHF11	Membrane associated ring finger 11
SEPT1	Sep-01	SEPTIN1	Septin 1
SEPT2	Sep-02	SEPTIN2	Septin 2
SEPT3	Sep-03	SEPTIN3	Septin 3
SEPT4	Sep-04	SEPTIN4	Septin 4
SEPT5	Sep-05	SEPTIN5	Septin 5
SEPT6	Sep-06	SEPTIN6	Septin 6
SEPT7	Sep-07	SEPTIN7	Septin 7
SEPT8	Sep-08	SEPTIN8	Septin 8
SEPT9	Sep-09	SEPTIN9	Septin 9
SEPT10	Sep-10	SEPTIN10	Septin 10
SEPT11	Sep-11	SEPTIN11	Septin 11
SEPT12	Sep-12	SEPTIN12	Septin 12
SEPT14	Sep-14	SEPTIN14	Septin 14
SEP15	Sep-15	SELENOF	15 kDa selenoprotein

### Converting dates which are mapped to more than one gene

**Figure 3:**
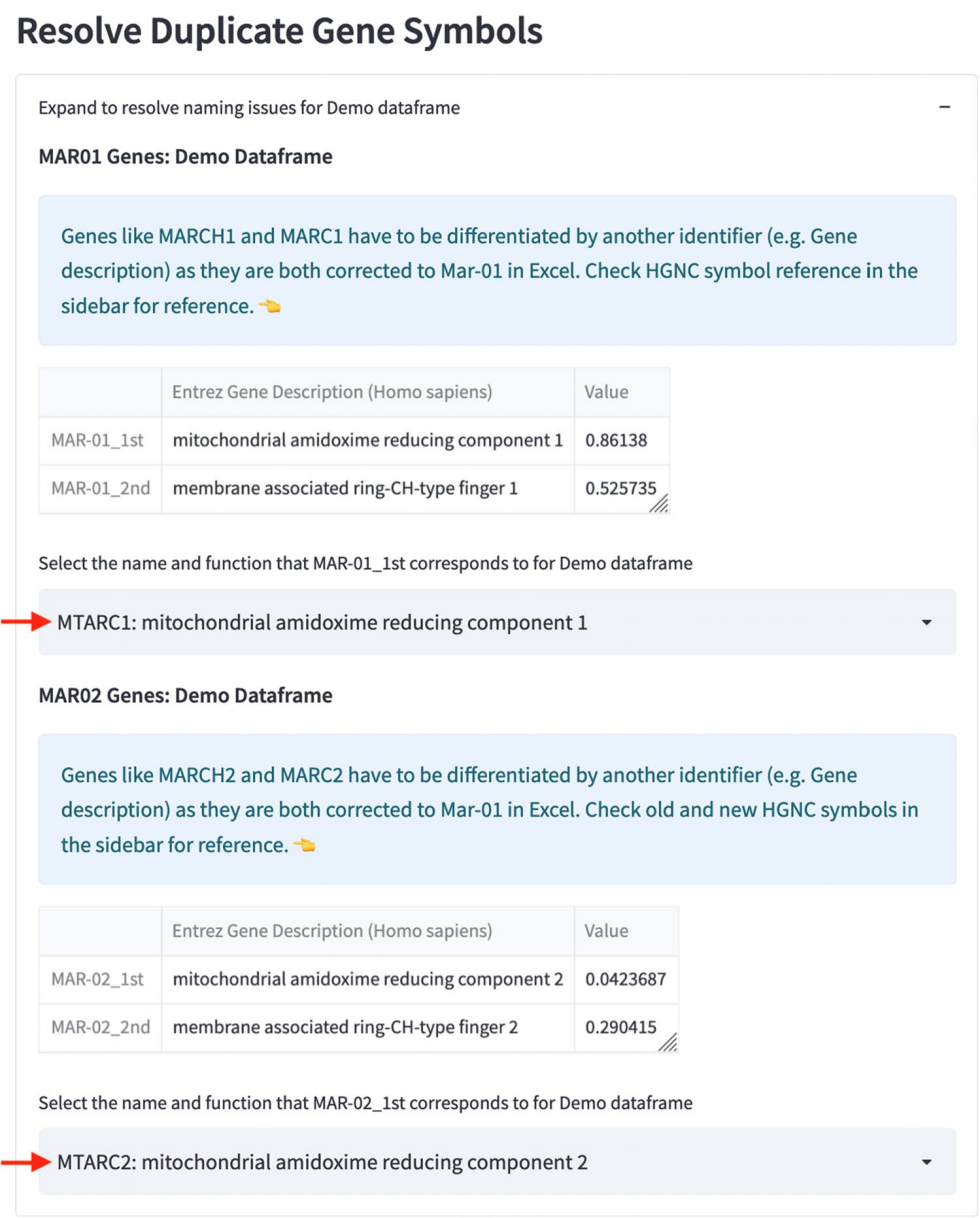
Gene Updater dashboard which is used to resolve duplicate gene symbols. Red arrows indicate the dropdown widgets that allows users to assign the correct gene name for Mar-01 and Mar-02.

To resolve gene duplicates related to Mar-01, Gene Updater converts the first instance of Mar-01 to Mar-01_1st and the second instance to Mar-01_2nd. Using the dropdown widget, users can assign the Mar-01_1st and Mar-01_2nd based on gene description or by any other unique identifiers (Fig. 3). The same procedure is then repeated for duplicates related to Mar-02. After the conversion of these dates, the output dataframe or file should have gene names that are updated to the new HUGO gene names.

### Output

The converted dataframe or file can be inspected within the Gene Updater web tool. Users can use the multi-query search bar to verify that the gene names are successfully updated. The output file with the updated gene names can then be exported as an Excel file.

### Performance of Gene Updater in autocorrecting Excel misidentified gene names

To evaluate the utility of Gene Updater in resolving Excel misidentified gene names, we leveraged on Mark Ziemann’s dashboard (http://ziemann-lab.net/public/gene_name_errors/Report_2022-05.html#journals-affected) to extract text and Excel files from various journals, including BMC Genomics, Nature, Genome Biology, Nucleic Acids Research, Human Molecular Genetics, BMC Bioinformatics, Nature Communications, PLoS One, Genome Research, Genes Development and RNA that were published in June 2022 (Table 2). A total of 356 text and Excel files were found, of which 81 of these files contain gene terms or gene symbols. Notably, 28 (34.6%) of the files with gene symbols contained date-related errors, highlighting the significance of having a tool to correct for these misidentified date terms (Table 2). Gene Updater was able to autocorrect for the majority of these files (78.6%), except for the files which contain Mar-01 and Mar-02 terms, but did not provide an accompanying unique identifier such as gene description or gene information (Table 2, right most column). These findings highlight the importance and utility of Gene Updater in rectifying misidentified gene terms, and emphasise the need to include a gene description column to better resolve MARC1, MARCH1, MARC2 and MARCH2 gene terms.

**Table 2:** Journals publishing text or Excel files with date-related errors in June 2022.

Journal	Text/Excel files found	Text/Excel files with gene symbols	Total number of files with date-related errors	Files without MAR-01 or MAR-02 date terms (%)	Files with MAR-01 or MAR-02 date terms	
					With gene description column (%)	Without gene description column (%)
BMC Genomics	87	7	3	3 (100.0)	0 (0.0)	0 (0.0)
Nature	8	1	0	0 (0.0)	0 (0.0)	0 (0.0)
Genome Biology	8	7	0	0 (0.0)	0 (0.0)	0 (0.0)
Nucleic Acids Research	40	10	1	1 (100.0)	0 (0.0)	0 (0.0)
Human Molecular Genetics	14	7	0	0 (0.0)	0 (0.0)	0 (0.0)
BMC Bioinformatics	1	1	1	1 (100.0)	0 (0.0)	0 (0.0)
Nature Communications	143	41	18	6 (33.3)	7 (38.9)	5 (12.2)
PLoS One	55	7	5	4 (80.0)	0 (0.0)	1 (14.3)
Genome Research	0	0	0	0 (0.0)	0 (0.0)	0 (0.0)
Genes Development	0	0	0	0 (0.0)	0 (0.0)	0 (0.0)
RNA	0	0	0	0 (0.0)	0 (0.0)	0 (0.0)
Total	356	81	28	15 (53.6)	7 (25.0)	6 (21.4)

Number of files with and without MAR-01/MAR-02 genes are indicated, and the respective percentages presented in parentheses. Gene Updater web tool is able to rectify most datasets, besides those datasets with Mar-01 and Mar-02 terms but without gene description information (indicated on the right most column).

### Comparison with other existing web tools

Presently, the two tools that can potentially convert dates to gene names are Oct4th (https://oct4th.sandbox.bio) and Truke (http://maplab.imppc.org/truke/). However, Oct4th only works on gene data files that have not been manipulated and processed in Excel. Moreover, the tool is presently unable to convert to the updated gene names, which are more resilient to auto-conversion. Truke can potentially convert the date formats to gene names, but can only convert dates that are labelled in the dd/mm/yy format, and process files one at a time. In contrast, our Gene Updater Streamlit web tool can process multiple .csv and .xlsx files, and takes into account the different kinds of date formatting that are converted by Excel, thus allowing faster and more efficient processing of dates to genes as compared to other existing web tools.

## Discussion

The automatic conversion of gene names to dates is a problematic feature of Excel. Analysis of major journals revealed that approximately 50–100 research articles to date still have gene name errors reported monthly (http://ziemann-lab.net/public/gene_name_errors/Report_2022-05.html#journals-affected). The fastest way to spot these errors is by sorting the column of gene names in ascending order, and the gene symbols that are converted to dates will appear as numbers at the top of the column. Thereafter, users will often have to manually convert these dates to text by specifying the cell formatting within Excel, which can be tedious and error-prone. Moreover, if the genes are auto-converted to Mar-01 or Mar-02, it is challenging to interpret whether the genes are either MTARC1-2 or MARCHF1-2, which are known to have contrasting biological functions. This date-to-gene converter web tool hence allows researchers to convert either old gene names or dates to the updated gene names quickly and reproducibly. In addition, we have incorporated multiple checkpoints that allow users to inspect the data before and after conversion.

Streamlit is an open-source framework that allows developers to create and deploy web apps easily. As the codes are also publicly available on GitHub, developers can easily customise the codes to convert and update any terms of interest. Future development of omics analysis tools can also incorporate the Gene Updater web tool framework to convert the old gene names and dates to the new gene names before executing any downstream pathway analysis algorithms.

## Conclusion

In summary, we developed a publicly available, user-friendly and customisable web tool that converts old gene names and dates back into updated gene names. We strongly encourage the processing of gene expression datasets with this web tool before publication or data sharing, to mitigate the risk of date auto-conversion.

## Methods

### Pathway analysis

Gene terms annotated in Table 1 were used as the input data for pathway analysis. The genes were analysed against the Gene Ontology (GO) Biological Processes database^6,7^ using the Enrichr tool^2^. Adjusted p-values for each enriched pathway were obtained from the Enrichr tool analysis and the bar charts were constructed using Prism 9.3.1 software.

### Running Gene Updater in web browser

Gene Updater is available to everyone and the running instance of the app can be located at https://share.streamlit.io/kuanrongchan/date-to-gene-converter/main/date_gene_tool.py. The documentations and instructions for use are made available within the Gene Updater app, and in this scientific publication.

### Running Gene Updater locally

To run the Gene updater locally, we have made the required codes, files, detailed instructions and technical requirements available within the web browser version of the Gene Updater app at: https://github.com/kuanrongchan/date-to-gene-converter (Zenodo; https://doi.org/10.5281/zenodo.6845701).

Briefly, Streamlit and Python 3.7 (or later) together with several python packages (pandas, numpy, regex, inflect, dateparser, xlrd, openpyxl, XlsxWriter, and steamlit-tags) with compatible versions should be installed locally. The following files should also be copied from our Gene Updater tool’s Github repository: (1) date_gene_tool.py, (2) requirements.txt, (3) demo.csv and (4) hgnc-symbol-check2.csv. For simplicity, users can also opt to download all the required files as a ZIP file within the GitHub repository.

After specifying the directory and folder with the downloaded files in terminal using the change directory (cd) command, users can then simply type: *streamlit run date_gene_tool.py*. This will generate a new tab with the Gene Updater web tool appearing in the default browser.

### Statement

All experiments and methods were performed in accordance with relevant guidelines and regulations.

## Data Availability

The demo dataset was created by us and contains Excel converted gene terms on the first column, gene description on the second column and random numeric values in the third column to allow the users to explore the features of the web tool without needing to upload any datasets. The demo dataset analysed in the current study is available in the GitHub repository, https://github.com/kuanrongchan/date-to-gene-converter (Zenodo; https://doi.org/10.5281/zenodo.6845701). Users can also use the Gene Updater web tool to analyse the demo dataset which is made available at: https://share.streamlit.io/kuanrongchan/date-to-gene-converter/main/date_gene_tool.py.
